# Unlocking the Bioactive Potential of Pomegranate Peels: A Green Extraction Approach

**DOI:** 10.3390/antiox12101796

**Published:** 2023-09-23

**Authors:** Giorgio Grillo, Giorgio Capaldi, Kristina Radošević, Željko Jakopović, Ksenija Markov, Mladen Brncic, Lorenzo Gallina, Emanuela Calcio Gaudino, Giancarlo Cravotto

**Affiliations:** 1 Department of Drug Science and Technology, University of Turin, Via P. Giuria 9, 10235 Turin, Italy; giorgio.grillo@unito.it (G.G.); giorgio.capaldi@unito.it (G.C.); lorenzo.gallina@unito.it (L.G.); emanuela.calcio@unito.it (E.C.G.); 2 Laboratory for Cell Cultures, Applications and Biotransformations, Department of Biochemical Engineering, Faculty of Food Technology and Biotechnology, University of Zagreb, Pierottojeva Ulica 6, 10000 Zagreb, Croatia; kristina.radosevic@pbf.unizg.hr; 3 Laboratory for General Microbiology and Food Microbiology, Department of Biochemical Engineering, Faculty of Food Technology and Biotechnology, University of Zagreb, Pierottojeva Ulica 6, 10000 Zagreb, Croatia; zjakopovic@pbf.hr (Ž.J.); kmarkov2@pbf.hr (K.M.); 4 Department of Food Engineering, University of Zagreb, Pierottijeva 6, 10000 Zagreb, Croatia; mbrncic@pbf.hr

**Keywords:** pomegranate peels, green extraction, food-waste valorisation, polyphenols, antioxidant activity, flavonoids, anthocyanins, green metrics, energetic evaluation, biological activity

## Abstract

Pomegranate (*Punica granatum* L.) is well known for its high content of bioactives, including polyphenols, flavonoids, and tannins, which have been shown to exhibit a wide range of biological activities, such as antioxidant, antimicrobial, and anticancer effects. It is worth noting that the majority of these molecules are found in the peels, which are usually disposed of after processing, causing a significant amount of waste, amounting to more than 3.6 million t/y. This work investigates microwave-assisted extraction (MAE) in water for the recovery of antioxidants from pomegranate peels (PP), including the optimisation of temperature and extraction times. The total phenolic, anthocyanin, flavonoid, and tannin contents of the recovered extracts were determined, as well as their antioxidant activities, which were found to be 356.35 mgGAE/gExtr, 303.97 µgCy3G/gExtr, 37.28 mgQE/gExtr, 56.48 mgGAE/gExtr, and 5.72 mmolTE/gExtr, respectively (according to the adopted reference). All results were compared with those obtained using a conventional protocol. In addition, the potential for water recycling by means of downstream nanofiltration in optimised MAE was investigated, leading to overall water reuse of approx. 75%. Power consumption (20.92 W/mgGAE) and common green metrics, Reaction Mass Efficiency (RME), E-Factor, and the Process Mass Intensiti/efficiency (PMI, PME), were considered in evaluating the proposed PP valorisation strategy. Finally, the biological activities of the main products were assessed. The antimicrobial properties of the PP extracts against three Gram-positive and three Gram-negative bacteria and their antiproliferative activity towards human cancer cells were tested. *S. aureus* bacteria was the most susceptible to the PP extracts. All tested products displayed antiproliferative activity against HeLa cells when higher concentrations were tested, with D-PP/NF (obtained from dried PP and sequential nanofiltration) being the most effective. This result was also confirmed via clonogenic analysis, which generally indicated the possible anti-cancer activity of pomegranate peel extracts obtained using this green approach.

## 1. Introduction

According to the FAO, approximately one-third of the world’s food production is lost or wasted over the various stages of the food supply chain. The extent of this loss varies greatly depending on the region and type of food. For fruits and vegetables, losses throughout the entire supply chain can be as high as 50%. This issue stands firmly among one of the United Nations’ Sustainable Development Goals (SDGs), which aims to reduce food waste by approximately 50% by 2050 [1]. 

This ambitious goal can be pursued using the circular economy concept, identified as a key principle for sustainable innovation, with an emphasis on creating a “zero waste” society and economy that aim to reduce waste and promote the efficient use of resources. In particular, (food)waste valorisation refers to the process of utilising this waste as a resource to create new products, rather than simply discarding it. Thus, it is necessary to emphasise that food waste, in addition to its applications in the energy, agronomy, and animal feed sectors, can provide a valuable feedstock for the recovery of bioactive compounds, including antioxidants and dietary fibre [2,3,4,5]. Extracting these compounds from food waste not only provides a sustainable approach to waste management but also generates value-added products that can be utilised in various applications, including as functional foods, nutraceuticals, and cosmetics [6,7]. Furthermore, the residues generated after food waste extraction for the recovery of valuable compounds can be further converted into new products, such as fermentation substrates [8], bioplastics [9], fine chemicals [10], or used as a source of energy through anaerobic digestion [11,12].

However, the extraction and conversion processes must be optimised to ensure that high-quality bioactive compounds are recovered while green chemistry principles are fulfilled. The importance of green chemistry in influencing future industrial processes cannot be denied. However, a significant number of procedures that involve extractions and chemical processes still rely on conventional reactors and methods [13]. The fundamental principles of green extraction focus not only on minimising waste, but also on optimising process efficiency while at the same time mitigating risks to human health and the environment [14]. This approach to so-called “process intensification” can be mainly achieved using enabling technologies such as microwave (MW), ultrasound, pulsed electric fields, supercritical fluid extraction, ohmic heating, hydrodynamic cavitation, etc. that aim to maximise process heat and mass transfer, leading to increased yield/conversion rates and, thus, savings in time and energy [15,16]. In this context, water-based extractions play a pivotal role, with water being considered “green” by definition as it is an easily usable and non-hazardous solvent [17]. Water, with its high polarity, possesses the ability to dissolve a wide range of polar compounds. However, although certain molecules (i.e., polyphenols) exhibit limited polarity, using water under subcritical conditions is still a viable option. Significant physico-chemical changes occur under these conditions, and selecting the appropriate temperature can lead to the recovery of several classes of target compounds [18].

Green metrics are key tools in assessing the environmental impact of chemical processes. They provide quantitative measures that evaluate the efficiency and sustainability of chemical reactions and processes. Reaction Mass Efficiency (RME), the E-factor, and Process Mass Intensity (PMI) are three crucial and commonly used green metrics [19].

RME is a metric that quantifies the use of reactants in a chemical reaction, so a higher RME value indicates more efficient use of raw materials and less waste generation. This parameter, which is typical for organic synthesis, can be adapted for extraction processes (although lower values are expected) [20]. The E-factor or environmental factor is a measure of the waste generated during a process: a low E-factor means a more sustainable process with minimal waste generation [21]. PMI is a metric that evaluates the efficiency of a chemical process by considering the overall mass of all materials, including reactants, solvents, catalysts, and products, per unit of desired product. A lower process mass intensity indicates a more resource-efficient process, as this indicates lower material consumption and waste generation. Another way to express PMI is the Process Mass Efficiency (PME), which is directly proportional to the sustainability of the overall process (see Section 2.7) [22]. These green metrics play a crucial role in the design and optimisation of sustainable processes. With the help of green metrics, the chemical industry can strive for more sustainable practices, minimise waste, conserve resources, and reduce the overall environmental footprint of chemical processes.

This study, investigating a new sustainable approach for the valorisation of pomegranate peels (PP) as a food residue using microwave-assisted extraction (MAE) with water as the solvent medium, fits well into this context.

Pomegranate (*Punica granatum* L.) is one of the world’s oldest known fruits, is deeply embedded in the cultures of the Mediterranean region, and is widely cultivated in many countries owing to its numerous health benefits [23]. Its production is estimated to be about 8.1 million tons worldwide, with Turkey being the fourth-largest producer after India, China, and Iran [24]. The fruits contain bioactive compounds such as polyphenols, flavonoids, and tannins, which have been shown to exhibit a range of biological activities, including antioxidant, anticancer, and antimicrobial effects [25,26,27,28,29].

However, the majority of these bioactive compounds are found in the peels and seeds, which are usually discarded during the processing of pomegranate fruits, leading to significant waste and environmental concerns. PP are generated during processing of the fruits, which involves the removal of the outer skin and separation of the seeds and juice. The generated quantity depends on the processing method and source of the fruit, but typically ranges from 30% to 50% of the total fruit weight [30]. We can therefore estimate, considering the abovementioned global production of the fruit, that around 3.6 million tons of waste are produced per year. This enormous amount of residue presents environmental and health risks, as it contributes to environmental pollution and disposal issues, particularly if not managed properly. Finding appropriate methods for the extraction of bioactive compounds from pomegranate residues and their transformation into added-value products is essential.

Many efforts have been undertaken to exploit the nutraceutical properties of the fruits, even leading to industrial applications, patents, and trademarks [31,32]. However, in recent years, there has been increasing interest in the recovery of bioactive compounds from residues and, in particular, from PP, using non-conventional technologies including MAE, ultrasound-assisted extraction (UAE), supercritical fluid extraction (SFE), and enzyme-assisted extraction (EAE), among others [33,34,35]. These innovative extraction technologies have shown great potential in improving the efficiency of the recovery of bioactives from biomass waste while reducing solvent usage and processing times. A recent review by Cano-Lamadrid et al. (2022) focused on the valorisation of pomegranate by-products and outlined several sustainable approaches to PP extraction, of which MW emerged as being particularly promising [33]. However, the reviewers concluded that: “although there are relevant and promising results, they are non-unanimous and scarce. Therefore, more research on MAE and comparison with other green techniques is required.” The present work, therefore, addresses this particular issue and aims to develop a microwave-assisted, sustainable, and efficient recovery of bioactives from PP. In particular, water extraction under MW was used, optimising both temperatures and extraction times. The developed strategy could lead to potential applications in the food, pharmaceutical, and nutraceutical industries. In particular, the total phenolic, anthocyanin, flavonoid, and tannin contents of the obtained PP extracts as well as their antioxidant activity (DPPH· and Cu chelation) were determined, and the results were compared with those of extracts obtained using the conventional protocol (hydroalcoholic solution). Finally, the biological activities of the PP extracts were investigated, in terms of antibacterial activity toward Gram-positive and Gram-negative bacteria as well as antiproliferative activity against a HeLa cell line.

In addition, in order ensure that the protocol was in line with the requisites of sustainable and eco-friendly protocols, a preliminary evaluation was performed on the optimised MAE for potential water recycling from the aqueous PP extracts, using membrane filtration via a nanofiltration protocol. This downstream strategy may not only enable water reuse for further extraction cycles but also concentrate the extracted antioxidants and dramatically reduce processing times and required power. To further support the proposed MAE procedure for PP valorisation, the produced results were also evaluated in terms of energy consumption and by means of the main green metrics, RME, E-Factor, and PMI (PME).

## 2. Materials and Methods

### 2.1. Pomegranate Peels and Chemicals

The PP (*Punica granatum* L. cv. ‘Wonderful’) utilised in this study were sourced fresh from O.P. La Deliziosa Soc. Coop. Agr. (Catania, Italy), distributed by Tropical Food Machinery (Parma, Italy). They were stored under frozen conditions at −20 °C and protected from light. A series of tests were conducted on the fresh peels, while a separate fraction was subjected to drying at 45 °C for a duration of 12 h in a ventilated laboratory oven (FALC Instruments, Treviglio, Italy). In order to determine the moisture content and proportions of organic and inorganic fractions, a thermogravimetric investigation was carried out using a muffle furnace (Nabertherm GmbH, Lilienthal, Germany). The experimental protocol involved dehydration at 100 °C for 12 h, followed by calcination at 650 °C for 4 h (see Table 1). All solvents and reagents utilised in this study were purchased from Sigma-Aldrich (St. Louis, MO, USA).

### 2.2. MW-Assisted Extraction (MAE) of Pomegranate Peels in Water

The MAE experiments were conducted using an MW multimodal reactor (SynthWAVE, Milestone, Bergamo, Italy) equipped with external inert gas feeding capability (N_2_). The optimisation of the extraction protocol was performed using 1 g of either fresh pomegranate peels (F-PP) or dried pomegranate peels (D-PP) with a solid-to-liquid ratio of 1:30. Lower ratios were excluded due to stirring difficulties, which could lead to undesired hot spots and matrix degradation. The screening explored three different extraction temperatures (100 °C, 125 °C, and 150 °C) with a MW power of 1500 W and three different time intervals (10, 20, and 30 min). Agitation was provided by a magnetic stirrer at ca. 650 rpm. In order to minimise oxidative stress in the biomass, each test was preceded by three N_2_ purges to eliminate atmospheric oxygen. Subsequently, the reaction chamber was pressurised with an appropriate amount of N_2_ to prevent water ebullition (5–10 bars). Following optimisation of the protocol, a preliminary scaled-up experiment was conducted using 20 g of biomass and 600 mL of solvent in a 1 L Teflon vessel, which were mixed using a mechanical stirrer at 650 rpm. The extraction process was performed at 100 °C for 10 min, utilising both fresh and dried peels. Furthermore, the same conditions were employed for the extraction of D-PP, utilising the permeate recycled from membrane nanofiltration.

After extraction, the resulting solutions were subjected to vacuum filtration after centrifugation at 4200 rpm to separate the solid particles. The dry extracts were subsequently recovered after freeze drying (LyoQuest-85, Telstar, Madrid, Spain), weighed, and stored at 4 °C for further analysis. Each run was performed in triplicate.

### 2.3. Hydroalcoholic Extraction of Pomegranate Peels

A conventional hydroalcoholic extraction was chosen as the benchmark protocol for pomegranate peels, for use as a comparison with the principal results achieved by means of MAE. The method involved reflux conditions using a hydroalcoholic solvent composition of 70% ethanol and 30% distilled water. A 5 g sample of dry peels was subjected to extraction with 150 mL of hydroalcoholic solution, resulting in a solid-to-liquid ratio of 1:30. The extraction was carried out for 1 h. Reflux conditions were achieved by employing an oil bath equipped with a magnetic stirrer (650 rpm). After extraction, the resulting solution was subjected to centrifugation at 4200 rpm to separate the solid particles, and it was then filtered under vacuum. The ethanolic fraction was removed using a rotavapor system operating at 40 °C and 175 mbar. The residual water fraction was removed under freeze-drying using a LyoQuest-85 freeze dryer (Telstar, Madrid, Spain). The dried extracts were weighed and stored at 4 °C for further analysis. Each run was performed in triplicate.

### 2.4. Membrane Nanofiltration (NF) for Bioactives Concentration and Water Recovery

Membrane nanofiltration (NF) was conducted on a 2 L solution of the D-PP extract produced under the optimised conditions (solid/liquid ratio of 1:30, 100 °C, 10 min). During this study, a pilot membrane filtration skid was used (PB100, Hydro Air Research Srl, Lodi, Italy). The system was equipped with a DKU 1812 NF membrane (filtering area: 0.38 m^2^, molecular weight cut-off range: 150–300 Da). During the process, the retentate was recirculated within the system while the permeate was continuously separated and collected, working under appropriate counter pressure (5 bars). The separation was performed for approximately 25 min. As a result, 500 mL of retentate and 1500 mL of permeate, which were freeze-dried, weighed, and stored at 4 °C for future analysis, were obtained from the 2 L solution. Part of the permeate was used “as obtained” for PP MAE in order to evaluate its recyclability in the process.

### 2.5. Colorimetric Tests on Pomegranate Peel Extracts: Quali-Quantitative Characterisation

#### 2.5.1. Total Phenolic Content (TPC)

The determination of total phenolic content (TPC) followed a modified version of the *Folin–Ciocalteu* procedure described by Hillis and Swain [36]. For each extract, an aliquot of 0.25 mL was combined with 0.5 mL of a 10% *w*/*v* Na_2_CO_3_ solution, followed by the addition of 0.25 mL of *Folin–Ciocalteu* reagent. The resulting solution was immediately diluted to a final volume of 5 mL using distilled water and thoroughly mixed. After allowing the solution to stand for 25 min, the absorbance was measured at 725 nm using a Cary 60 UV–vis spectrophotometer (Agilent Technologies, Santa Clara, CA, USA). A calibration curve was prepared using gallic acid solutions ranging from 0.01 to 0.45 mg/mL. TPC was expressed as mg/g of gallic acid equivalents (GAE) over the extract (referred to as TPC selectivity) and over the matrix (referred as TPC yield). Each test was performed in triplicate.

#### 2.5.2. Total Anthocyanin Content (TAC)

The total anthocyanin content (TAC) was determined using the pH differential method [37]. The dried extract was dissolved in deionised water to achieve a concentration of approximately 1.5 mg/mL. Two solutions were prepared by mixing 1 mL of the sample with 5 mL of potassium chloride (0.025 M KCl, pH 1 with HCl) and sodium acetate buffer (0.4 M CH_3_COONa, pH 4.5 with HCl). After allowing the solutions to equilibrate for 5 min, the absorbance was measured at 510 and 700 nm using a Cary 60 UV–Vis spectrophotometer (Agilent Technologies, Santa Clara, CA, USA). Deionised water was used as a blank. The resulting sample absorbance was calculated using Equations (1) and (2):

A = (A_λvis-max_ − A_700_)_pH 1.0_ − (A_λvis-max_ − A_700_)_pH 4.5_ (1)

Anthocyanin content (mg/L) = (A × MW × DF × 1000)/(ε × 1)(2)

where MW is the molecular weight and ε is the molar absorptivity of the anthocyanin pigment in an acidic aqueous solvent. The values used in this formula correspond to cyanidin-3-glucoside (Cy3G, MW = 449.2 and ε = 26,900), meaning that the TAC results were expressed as Cy3G equivalents. Each test was performed in triplicate.

#### 2.5.3. Total Tannin Content (TTC)

The determination of total tannin content (TTC) was performed using the Peri and Pompei protocol [38] with some adjustments, as reported by Aimone et al. [39]. In this analysis, 600 µL of hemisulfate cinchonine solution (0.5% *w*/*v*) was added to 600 µL of the extract solution in a 1.5 mL Eppendorf tube. The resulting mixture was shaken and then left overnight at 4 °C to promote the precipitation of tannate cinchonine. The sample was then centrifuged at 26,000 rpm for 2 min at 10 °C (Allegra 64R, Beckman Coulter Srl, Milano Italy), and the supernatant, which contained a polyphenol-rich solution, was separated and analysed using the *Folin–Ciocalteu* test (see Section 2.5.1). The total tannin content was calculated by determining the difference between the TPC of the fresh sample and the TPC obtained after tannin precipitation, and was expressed as gallic acid equivalents (GAE). Each test was performed in triplicate.

#### 2.5.4. Total Flavonoid Content (TFC)

The quantification of total flavonoid content (TFC) was performed using the colorimetric method described by Saikan Set et al., with modifications [40]. A 0.5 mL aliquot of the sample (water solution) with a concentration of 1 mg/mL was mixed with 0.1 mL of 10% aluminium nitrate, 0.1 mL of 1 M potassium acetate, and 4.3 mL of 80% ethanol. The mixture was thoroughly mixed and allowed to stand at room temperature for 40 min. The absorbance of the supernatant was measured at 415 nm (Cary 60 UV–Vis spectrophotometer, Agilent Technologies, Santa Clara, CA, USA) to determine the presence of flavonoids based on the development of a yellow color. The results were expressed as quercetinu equivalents in milligrams per gram (mgQE/g) of dry extract, using a standardised calibration curve. Each test was performed in triplicate.

#### 2.5.5. Total Sugar Content (TSC)

The quantification of total carbohydrate content (TSC) was conducted using the anthrone method, with modifications [41]. A calibration curve was prepared using a glucose–water solution, with dilutions ranging from 10 to 200 µg/mL, to serve as a reference for determining the sugar content in the samples. An aliquot of the sample solution, consisting of 1 mL with concentrations ranging from 0.01 to 0.5 mg/mL in deionised water, was mixed with 5 mL of anthrone reagent (0.2 g anthrone in 100 mL of concentrated 96% sulfuric acid). The solution was then heated at 100 °C for 20 min and the absorbance was measured at 620 nm (Cary 60 UV–Vis spectrophotometer, Agilent Technologies, Santa Clara, CA, USA). The increase in color intensity, from yellow to green/blue, was directly proportional to the carbohydrate concentration in the sample. The obtained data were analysed using a glucose standard calibration curve and expressed as glucose equivalents in milligrams per gram (mgGluE/g) of dry extract. Each test was performed in triplicate.

#### 2.5.6. Antioxidant Activity: DPPH· Inhibition

The antioxidant activity of the extracts was assessed using the method described by Brand-Williams et al. [42]. The antioxidant activity of the extracts was determined by measuring the inhibition of DPPH· radicals, which indicated the scavenging ability. The EC50 value, which represents the concentration of the extract required to inhibit 50% of DPPH· radicals, was determined as the parameter for scavenging activity. A Trolox hydroalcoholic solution (50% EtOH *v*/*v*) served as the standard reference. The sample EC50 values were then compared to the EC50 values obtained from the standard to express the results as Trolox equivalents (μmol TE/mL). Various dry extract concentrations were prepared via sequential dilution, and the absorbance was measured at 515 nm using a Cary 60 UV-Vis spectrophotometer (Agilent Technologies, Santa Clara, CA, USA). The obtained absorbance data were processed using Bobo Least Squares software (ver. 0.9.1.) with probit regression analysis [43]. Blank samples containing only water and methanol were used for instrument zeroing, while blank samples with the dry extract but without DPPH· radicals, were used to account for matrix effects. A reference sample containing water and DPPH· radicals was utilised to normalise the results and verify absorbance due to natural inhibition. Each test was performed in triplicate.

#### 2.5.7. Cu Chelating Activity of PP Extracts

The Cu chelating ability of the sample was evaluated using the pyrocatechol violet (PV) assay, following the method described by Santos et al. [44]. PV forms a dark red complex with Cu^2+^ ions not bound by polyphenols in a slightly acidic medium, and the rate of color reduction indicates the Cu chelating activity. A solution containing the extract was mixed with sodium acetate buffer (4.1 g/L, pH 6) and copper sulfate solution (50 mg/L), to which the PV solution (773 mg/L) was then added. After specific reaction times, the absorbance was measured at 632 nm using a Cary 60 UV-Vis spectrophotometer (Agilent Technologies, Santa Clara, CA, USA). The inhibition of PV-Cu^2+^ complex formation was expressed as the percentage of inhibition compared to a reference solution prepared without the extract. The percentage inhibition was calculated using different sample concentrations, and the EC50 value was determined using probit regression and Bobo Least Squares software [35]. The sample EC50 values were then compared to the EC50 values obtained from an EDTA solution to express the results as EDTA equivalents (μmol EDTA/mL). Each test was performed in triplicate.

### 2.6. Biological Activities

#### 2.6.1. Antibacterial Activity Test

Gram-negative bacterial strains, including *Escherichia coli* 3014, *Pseudomonas aeruginosa* 3024, and *Salmonella typhimurium* 3064, and Gram-positive bacterial strains, including *Staphylococcus aureus* 3048, *Bacillus subtilis* 3045, and *Listeria monocytogenes* 3112, were obtained from the collection of microorganisms at the Laboratory of General Microbiology and Food Microbiology, Faculty of Food Technology and Biotechnology, University of Zagreb (Zagreb, Croatia). All cultures were stored at −70 °C in nutrition broth (Biolife, Milano, Italy) with 30% glycerol (by volume). To perform the antibacterial activity test, microorganisms were firstly activated via incubation at 37 °C in the corresponding broths. The antibacterial activity of the PP extracts (F-PP, D-PP, and D-PP/NF scaled-up extracts and hydroalcoholic benchmark) was tested on selected bacterial cultures using disc diffusion assays. Bacterial cultures were prepared and the CFUs of all used suspensions were determined, amounting to 10^7^–10^8^ CFU mL^−1^. To perform the test, 100 µL of the bacterial suspension was smeared using a Drigalski rod onto the nutrient agar plate and left to dry. After 30 min, sterile filter discs (6 mm) (Macherey-Nagel GmbH, Düren, Germany) were immersed in 50, 250, and 500 µg/mL of the PP extracts and placed on the surface of the inoculated nutrient agar. After incubation for 24 h at 37 °C, inhibition was detected as the clear halo diameter (mm). Discs with chloramphenicol (10 µg per disc) (Liofilchem, Roseto, Italy) were used as positive controls, while discs immersed in the sterile water that was used to make the water solutions of the PP extracts were used as negative controls for all tested microorganisms. All assays were performed in triplicate.

#### 2.6.2. Antiproliferation Assay on Human Cell Line

The antiproliferative activity of the PP extracts (F-PP, D-PP, and D-PP/NF scaled-up extracts and hydroalcoholic benchmark) was evaluated in vitro against an adherent human tumor cell line using the CellTiter AQueous One Solution Cell Proliferation Assay (Promega, Madison, WI, USA). HeLa (ATCC No. CCL-2) cells were cultured in Dulbecco’s Modified Eagle’s Medium (DMEM) (Sigma, St. Louis, MO, USA) supplemented with 5% (*v*/*v*) heat-inactivated foetal bovine serum (FBS) (Gibco Invitrogen Corporation, Paisley, UK) and maintained in BioLite Petri dishes (Thermo Fisher Scientific, Waltham, MA, USA) in an incubator with a humidified atmosphere and 5% CO_2_ at 37 °C. The experiments were performed three times with four parallels for each concentration of extract tested in BioLite 96-well plates (Thermo Fisher Scientific) seeded with exponentially growing cells at the indicated concentration (∼3 × 10^4^ cells per well in 100 μL of medium) and incubated for 24 h. Lyophilised extracts were dissolved in 10 mg/mL of water and further diluted in DMEM to the final tested concentration (50, 100, 250, and 500 μg/mL) before being applied to the cells. The control cells were untreated cells. After treatment, the cells were incubated for an additional 72 h, after which the CellTiter AQueous One Solution Cell Proliferation Assay was performed according to the manufacturer’s instructions, with minor modifications. In brief, 10 μL of the CellTiter AQueous One Solution Cell Proliferation Reagent was added to each well and the cells were incubated for an additional 3 h, after which the absorbance was measured at 490 nm using a microplate reader (Tecan, Switzerland). Cell viability was expressed as the percentage of treated cells over control cells. The experiments were performed three times with four parallels for each concentration of tested extracts. The experimental results were statistically analysed using Statistica 8 software. Data in Section 3.3.2. are expressed as the mean ± standard deviation (±SD), indicated as an error bar. The differences between means were analysed using the ANOVA test, followed by a post-hoc Tukey’s test. A significant difference was considered at a *p*-value < 0.05.

#### 2.6.3. Clonogenic Analysis

The clonogenic assay began by seeding pre-cultured HeLa cells in 6-well plates at an initial concentration of 200 cells in 2 mL of culture medium per well. The cells were incubated under optimal conditions and treated with the scaled-up extracts of F-PP, D-PP, and D-PP/NF and the hydroalcoholic benchmark at a concentration of 500 µg/mL after 24 h. Untreated control cells were also analysed. Three days after the cells were treated, the growth medium containing the tested extracts was removed and replaced with fresh growth medium, after which the plate with HeLa cells was returned to the incubator for further cultivation. After treatment, the surviving cells required about 1–3 weeks to form colonies. In this work, the produced colonies were visible 19 days after the initial seeding of the cells. Staining the grown colonies with crystal violet was performed after removing the growth medium and washing the cells with 1 mL of PBS buffer. A 2.5 mL volume of methanol was then added to fix the cells, which was removed after 10 min. The plates were then allowed to air dry completely. A 0.5% solution of crystal violet was then added, and the cells were incubated for 10 min. In the final step, the dye was removed and the colonies in the wells were rinsed with 1 mL of PBS buffer and deionised water. The number of grown colonies was then counted and the plating efficiency (PE) and survival fraction (SF) were calculated according to the equations in the protocol reported by Franken et al. [45]. PE was the ratio of the number of colonies to the number of seeded cells, while SF was the number of colonies formed after the treatment of the cells, expressed as PE.

### 2.7. Green Metrics

The environmental impact of the proposed process was evaluated by means of three principal green metrics: RME, E-Factor, and PMI (expressed as PME%).

RME, which quantifies reactant exploitation, represents the proportion of the total mass of reactants converted into desired products (see Equation (3)).

(3) $$Reaction\;Mass\;Efficecny\;(RME,\;\%)=\frac{Mass\;of\;Product}{Mass\;of\;Matrix}\times 100$$

The E-factor measures the waste generated during a process and is calculated by dividing the total mass of waste generated by the mass of desired product (see Equation (4)).

(4) $$E-Factor=\frac{Total\;Mass\;of\;Waste}{Mass\;of\;Product}$$

PMI considers the overall mass of all materials, including reactants, solvents, catalysts, and products, per unit of desired product (see Equation (5)). It can also be expressed as a percentage, the PME, which is directly proportional to the overall process sustainability (see Equation (6)).

(5) $$Process\;Mass\;Intensity\;(PMI)=\frac{Total\;Mass\;in\;the\;Process}{Mass\;of\;Product}$$

(6) $$Process\;Mass\;Efficency\;(PME,\;\%)=\frac{1}{PMI}\times 100$$

## 3. Results and Discussion

### 3.1. MW-Assisted Extraction of Pomegranate Peels (PP): Lab-Scale

The MAE of PP was screened using water as the solvent at three different temperatures (100 °C, 125 °C, and 150 °C) for 10, 20, and 30 min. In order to explore the matrix effect, PP were extracted both in fresh (F-PP) and dry form (D-PP). The products were characterised in terms of dry yield, TPC, and TAC, and thus the best performing conditions were defined. The optimised protocol was then reproduced in a 1 L scaled-up protocol, the products of which were further characterised in terms of TFC, TTC, TSC, DPPH· inhibition, and Cu chelating activity (See Section 3.2). Biological activities were also assessed as antibacterial activity against Gram-positive and Gram-negative bacterial strains and antiproliferative activity toward a human tumor cell line (See Section 3.3).

#### 3.1.1. Dry Yield and Total Phenolic Content (TPC)

**Figure 1:**
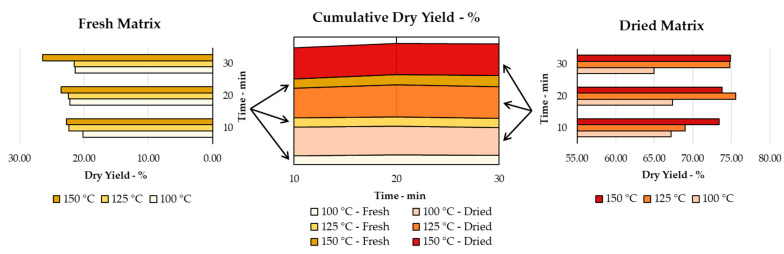
Dry extraction yields for MAE of F-PP and D-PP: time and temperature screening, with cumulative dry yield reported as stacked percentages for comparison.

The dry yields were normalised to the feeding matrix. This is important from a productivity point of view when considering the overall mass fed into the process. One of the aims of this work was to assess the exploitability of PP as a source of bioactives, also with a view to possible industrial use. An important point was, therefore, the amount of final product per amount of matrix used. The dry yields of each extract from the MW-assisted protocols depended on the temperature and process duration, for both F-PP and D-PP. Figure 1 reports the outcomes for each matrix together with the “cumulative dry yield,” with the percentages stacked to allow for direct comparison (See Table A1 for details). Thus, it is worth noting that D-PP (darker shades, larger areas) yielded higher recoveries than F-PP (lighter shades, smaller areas) over all of the time and temperature conditions explored. Moreover, at fixed temperature, it was possible to observe that time did not strongly affect the process, as limited yield fluctuations were seen.

In general, the best dried yields, for both F-PP (26.40%) and D-PP (74.85%), were achieved at 150 °C after 30 min of MAE, albeit with about 50% more extract being recovered from D-PP. However, comparable results were achieved for the latter (approx. 74.8%) at 125 °C after 30 min.

**Figure 2:**
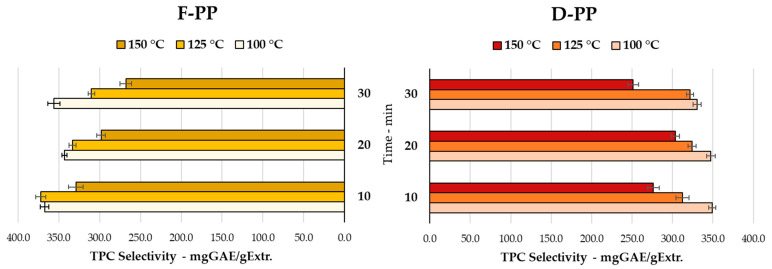
TPC selectivity for MAE of F-PP and D-PP: time and temperature screening.

To better understand the MAE process trends, the *Folin-Ciocalteu* assay was adopted to evaluate the TPC of each recovered extract, in terms of selectivity and yield (see Section 2.5.1). The first parameter, which expresses the amount of polyphenols (mgGAE) per gram of extract, is reported in Figure 2.

TPC selectivity displayed the opposite trend to that of dry yield: polyphenol ratios decreased as the temperature and treatment time increased. This behaviour, which was observed in both matrices, may have been related to the partial thermolability of the metabolites contained in the PP, together with an increase in non-phenolics co-extracts [46,47]. The latter may be explained by the salts and oligo- or polysaccharides that were partially hydrolysed and thus solubilised by the subcritical conditions, which became harsher the closer the process came to 150 °C [48,49]. This last hypothesis will be addressed further in the characterisation performed on the optimised scaled-up samples (up to 600 mL, See Section 3.2.1). In detail, 367.36 and 349.14 mgGAE/gExtr were achieved at 100 °C after only 10 min for F-PP and D-PP, respectively, whereas 267.90 and 251.30 mgGAE/gExtr were produced at 150 °C and 30 min. Unlike the dry extraction yield, the difference between the starting materials did not appear to be remarkable for TPC selectivity. In summary, TPC selectivity was favoured when restricting MAE screenings to low temperatures and limited processing times.

**Figure 3:**
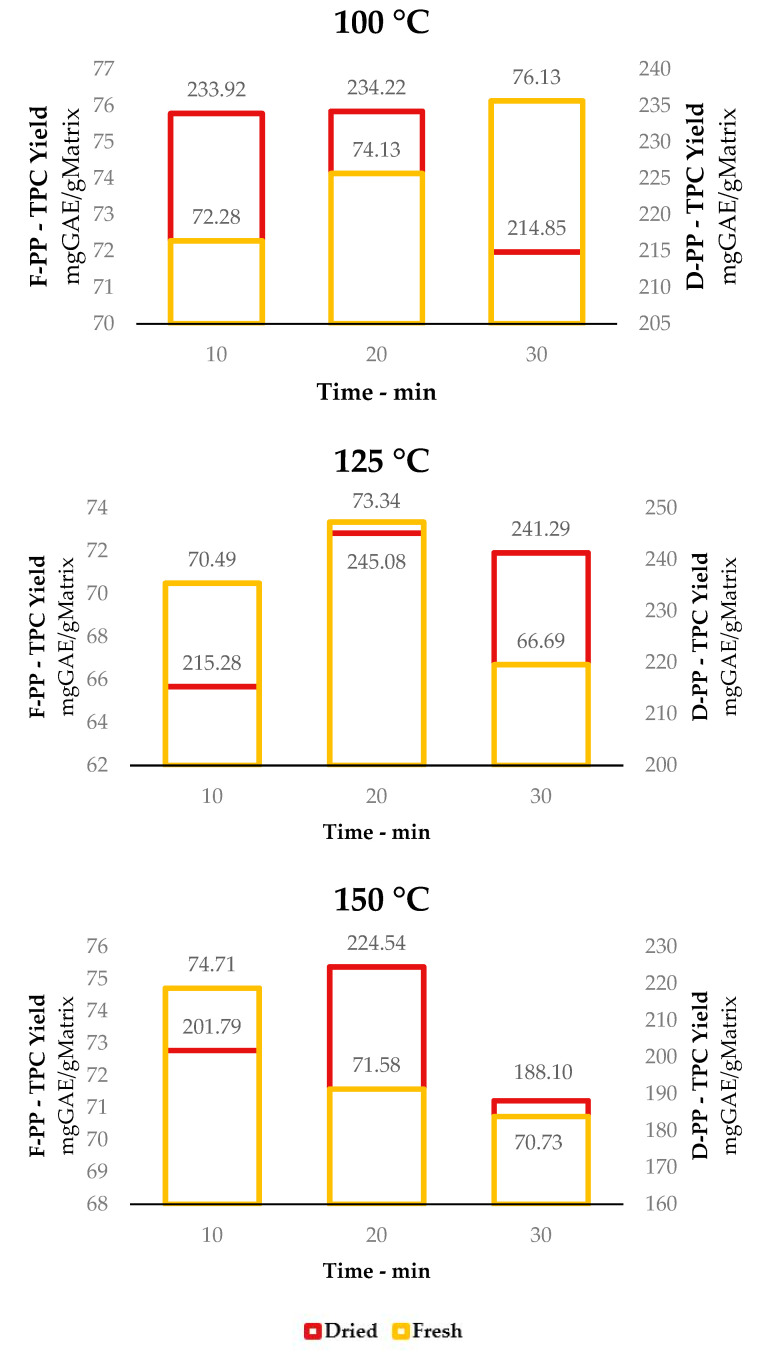
TPC yields for MAE of F-PP and D-PP at different temperatures: 100 °C, 125 °C, and 150 °C.

In order to better understand how using either fresh or dried PP could influence extraction procedure effectiveness, we moved to an evaluation of process productivity. For this purpose, TPC yield was determined as the parameter of choice as it can represent a combination of polyphenol selectivity and dry extraction yield (mgGAE/gMatrix). The results are depicted in Figure 3 (100 °C, 125 °C, and 150 °C, respectively).

As expected, the dramatic difference in the productivity of fresh and dried peels was clearly visible. The latter achieved up to 245.08 mgGAE/gMatrix, whereas 76.13 mgGAE/gMatrix was produced by F-PP, representing a 3-fold increase (125 °C, 20 min and 100 °C, 30 min, respectively). Here, the volumetric contraction caused by matrix dehydration had a clear effect. Thus, for a given amount of treated biomass, the quantity of the processed “organic” fraction was clearly enhanced, which had a clear positive effect on process productivity. The results of the TPC evaluations indicated that a general decrease in efficiency occurred during longer extractions (30 min), regardless of the process temperature, which seemed to only slightly affect the phenolic compounds. These data appeared to indicate that shorter and less harsh extraction conditions were to be preferred.

Once we had determined that D-PP provided the best productivity in terms of polyphenol recovery, this work was extended further to define whether the thermal dehydration step had an impact on two other aspects of the final product: the anthocyanin content and overall energy consumption (Section 3.1.2 and 3.2.2).

#### 3.1.2. Total Anthocyanin Content (TAC)

**Figure 4:**
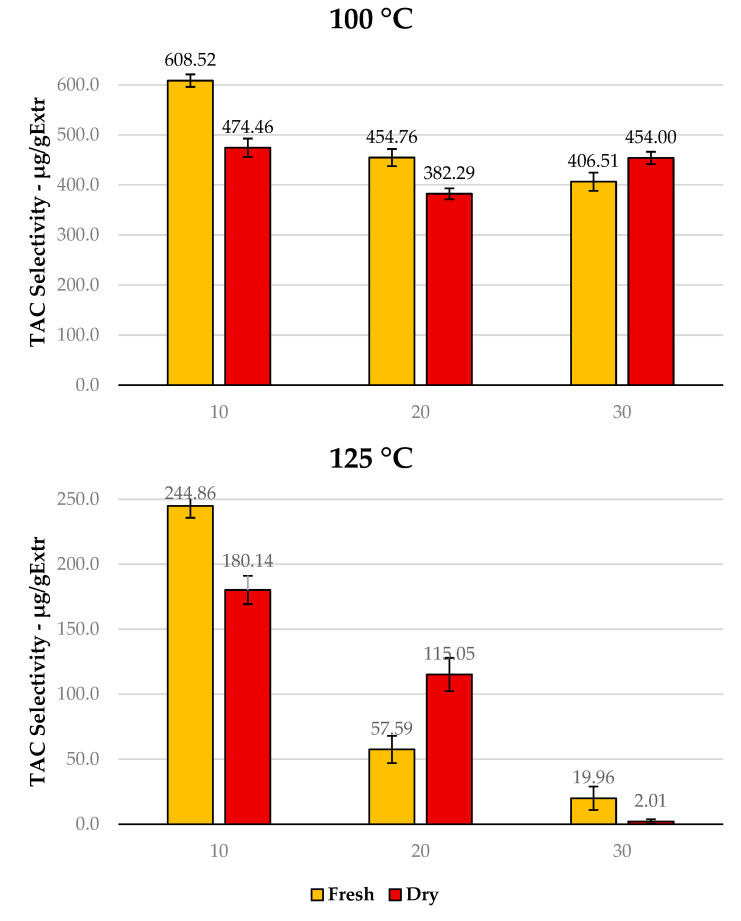
TAC seletcitivies for MAE of F-PP and D-PP: time and temperature dependence at 100 °C and 125 °C. 150 °C is not reported due to the absence of signal.

The first point in evaluating the effect of the drying process on PP valorisation was to determine the recovery efficiency towards the most thermolabile class of metabolites present in PP, anthocyanins. The colorimetric assay adopted for this screening (See Figure 4) revealed, as expected, inverse proportionality to MAE extraction temperature, which worsened even further at longer times (from 10 to 30 min). Accordingly, anthocyanins were completely absent in the product achieved at 150 °C. 

MAE at 100 °C for 10 min of F-PP led to a maximum TAC of 608.52 μgCy3G/gExtr, whereas D-PP produced 474.46 μgCy3G/gExtr, representing an approx. 1.5-fold increase in anthocyanin recovery. In general, it was possible to state that anthocyanins were not the main component available in the processed material, even in the fresh matrix. This could be extrapolated from the fact that, despite high TAC selectivity, the overall TPC yield was significantly lower for F-PP (72.28 mg GAE/gMatrix vs. 608.52 μgCy3G/gExtr) than for D-PP (233.92 mg GAE/gMatrix vs. 474.46 μgCy3G/gExtr). Hence, from this point of view, the drying step did not seem to excessively affect the quality of the recovered extract. It worth noting that this work was not strictly focused on anthocyanins, which, in addition to not being predominant in PP, tend to present highly fluctuating contents owing to biomass handling and storing conditions. Moreover, these metabolites require particular extraction conditions (i.e., acidic media) that could potentially affect the sustainability of the whole valorisation protocol and go beyond the approach proposed in this manuscript.

### 3.2. MW-Assisted Extraction of Pomegranate Peels (PP): Scale-Up

This work aimed to design a valorisation strategy for pomegranate residues by means of metabolite extraction in water under MAE. This ambitious task could not ignore the need for a scaled-up approach because of the enormous amount of generated waste. For this reason, the second part of this manuscript is dedicated to the exploitation of a 1 L MW-assisted extraction system, preliminary tackling this point. This study could pave the way for further industrial applications, as MW has reached the appropriate technological maturity (TRL) to be a viable option for large-scale production purposes, including biomass valorisation [50,51].

The process set-up was defined by merging the results of the lab-scale screenings, which indicated 100 °C and 10 min to be the best performing conditions for maximum bioactive recovery. Both fresh and dried peels were tested for the sake of comparison. 

#### 3.2.1. Extract Characterisation

**Figure 5:**
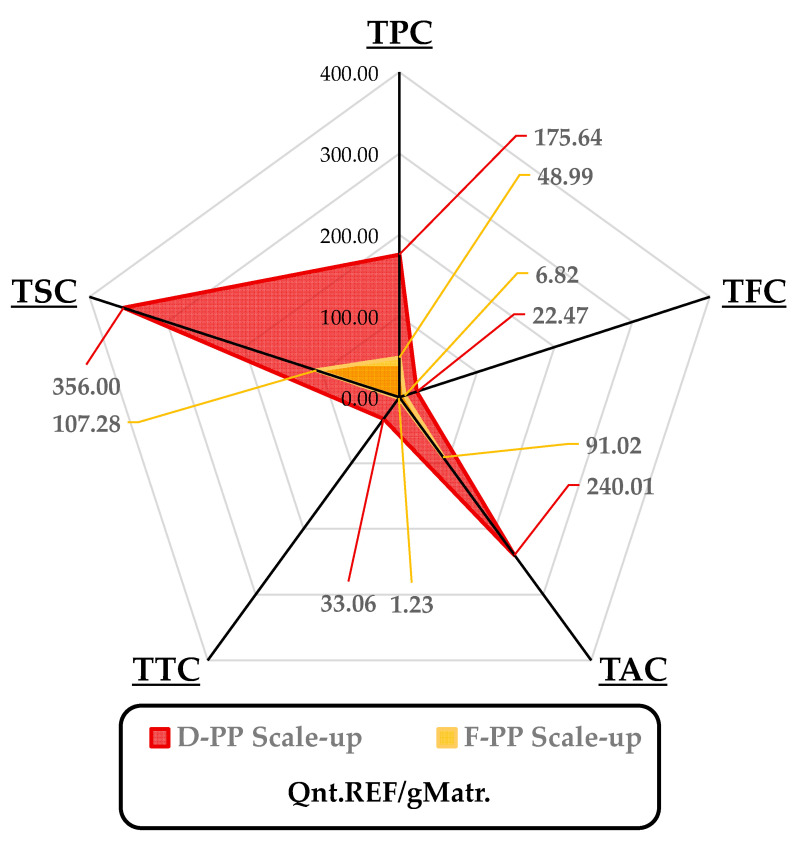
The main components of the quali-quantitative analysis for MAE scale-up of F-PP and D-PP. Yields are reported per gram of extracted matrix, reporting total weights of polyphenols, flavonoids, anthocyanins, tannins, and sugars according to the adopted reference. TPC: mgGAE; TFC: mgQE; TAC: µgCy3G; TTC: mgGAE; TSC mgGLU.

Taking process intensification as the main focus, the extracts achieved by means of MAE scale-up were characterised using TPC and TAC assays, together with additional TFC, TSC, and TTC tests and an evaluation of preliminary antioxidant activity, in terms of radical inhibition (DPPH·) and Cu chelating capability (See Figure 5 and Table 2).

The analysed parameters aligned with the trends observed in the lab-scale protocol, wherein metabolite selectivity did not substantially differ between F-PP and D-PP, whilst the corresponding yields, as depicted in Figure 5, were more than 3 times higher for the dried PP matrix, except for the anthocyanin content. 

**Figure 6:**
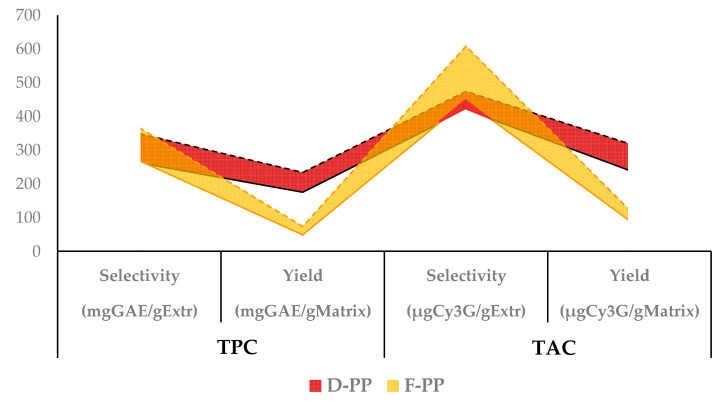
Comparison of TPC and TAC variations in scale-up transposition. Selectivities and yields are reported for D-PP and F-PP. Dashed lines: Lab-scale; Solid lines: Scaled-up.

Furthermore, a direct comparison of the lab-scale and scaled-up approaches (see Figure 6) made it possible to evaluate the trends in the main analysed components: TPC and TAC (first screenings in Section 3.1.1 and Section 3.1.2, respectively). When a higher amount of matrix was processed, these two parameters registered a contraction in both selectivity and yield of approx. an average of 1.4-fold. It is worth noting that a minimal performance drop could be assumed to be physiological when transposing a process towards higher volumes owing to the impossibility of achieving a completely “geometrical” scale-up. The up-scaling of stirred vials/vessels/tanks leads to large differences in geometric properties at the laboratory and pilot scales (especially at higher scaling factors), so that it is usually difficult to achieve complete geometric and procedural similarity. It could be excluded that the abovementioned differences were related to MW penetration, as the tested volumes were within the reactor application range, but rather were the result of mass transfer variations. However, this phenomenon could certainly be reduced/eliminated by adjusting the system accordingly.

As expected, greater variability in TAC could be observed in F-PP owing to its higher content of these metabolites. Similarly, this same matrix showed the smallest yield reductions (for both TPC and TAC), caused by the limited amount of extracted plant material owing to the high water content. Conversely, D-PP showed a more stable trend, with limited losses for all of the explored parameters.

From this point onward, the scaled-up aqueous MAE protocol was explored and characterised further using both F-PP and D-PP for the sake of comparison.

Polyphenolic compounds act as potent scavengers of reactive oxygen and nitrogen species (ROS and RNS, respectively), as well as metal-chelating agents. Accordingly, these metabolites can be exploited for the prevention of mutation-related diseases owing to their antioxidant features, [51] which act dynamically in the balance between direct ROS/RNS quenching and metal chelating capacity [52,53].

The inhibition of DPPH· radicals and the Cu chelation features were investigated to evaluate extract antioxidant activity, as depicted in Table 2.

No pronounced differences in the antioxidant and Cu chelating powers of the two pilot scale PP extracts were detected (Table 2), although F-PP displayed better antioxidant power than D-PP (13.18 vs. 5.72 Trolox eq.), while the latter exhibited slightly higher Cu chelating power (529.81 vs. 513.66 EDTA eq.). Although F-PP displayed double the antioxidant potential in the DPPH· essay, both extracts exhibited remarkably high activity at concentrations of a few micrograms per millilitre. Thus, a deeper investigation using a biological assay was conducted (details can be found in Section 3.3).

#### 3.2.2. Fresh and Dried Pomegranate Peels: Power Consumption

So far, all of the collected data confirmed that MAE of PP (100 °C, 10 min) provided a valuable extract in terms of the recovered metabolites and their antioxidant activity. The previous comparisons aimed to define which matrix could be suitably exploited in a valorisation protocol. For this purpose, an additional evaluation, power consumption, was performed to assess the impact of the extraction and dehydration steps on TPC yield. Polyphenols were adopted as the reference compounds as they were the most comprehensively available parameter. The elements used to calculate Polyphenol Power Efficiency, expressed as W/mgGAE, are reported in Table 3.

It is worth noting that although the dehydration step in producing D-PP required extra power intake (approx. 0.98 kW), the enhanced TPC yield (175.64 vs. 48.99 mgGAE/gMatrix) meant that the resulting *Polyphenol Power Efficiency* for D-PP was 11.07 W/mgGAE, which was a Watts savings of more than 55% per mgGAE compared to F-PP. 

Thus, considering the characterisation profile and energy required to recover the extract, D-PP was more suitable as a feedstock for valorisation by means of MAE, considering the productivity enhancement related to the dehydration step.

#### 3.2.3. Nanofiltration: Water Recovery and Metabolite Concentration

The extract produced via the optimised MAE of D-PP (100 °C, 10 min) was considered for membrane filtration using a nanofiltration protocol (NF, 150–300 Da). This downstream strategy leads to the recovery of large quantities of water, while also concentrating the final active product at the same time, dramatically reducing the need for time- and energy-consuming work-up procedures. 

**Figure 7:**
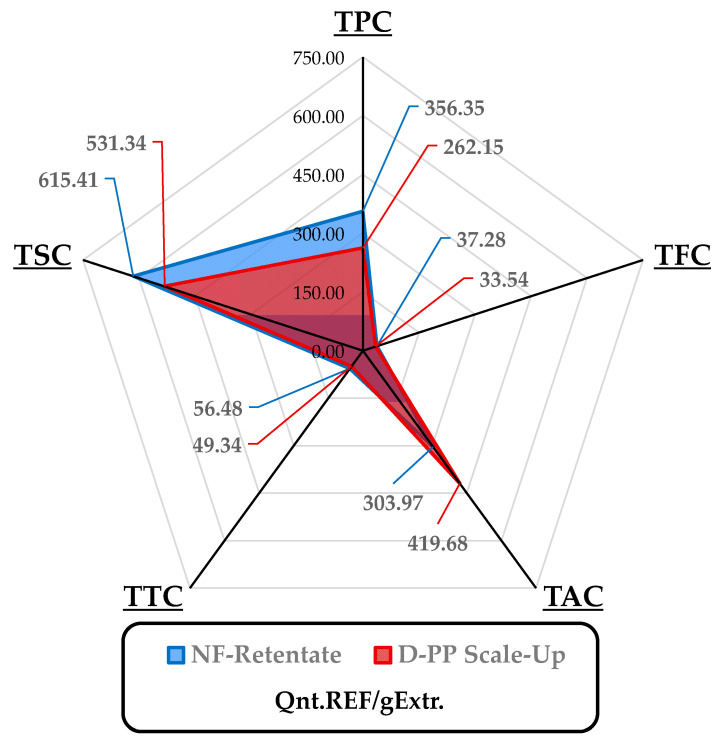
Main components of quali-quantitative analysis for MAE scale-up of D-PP and its NF retentate. Selectivity is reported per gram of extract, reporting total weights of polyphenols, flavonoids, anthocyanins, tannins, and sugars according to the adopted reference. TPC: mgGAE; TFC: mgQE; TAC: µgCy3G; TTC: mgGAE; TSC mgGLU.

NF was performed on 2 L of D-PP extract mixture using a pilot membrane filtration system (PB100, Hydro Air Research Srl) equipped with a DKU 1812 NF membrane (filtering area: 0.38 m^2^, cut-off range: 150–300 Da) that collected the concentrated metabolites in the retentate and the removed water in the permeate stream. The extract was concentrated 3.3 times (from 18.99 to 62.86 mg/mL) after 25 min of processing, with an overall water recovery of approx. 75%. In addition, NF treatment partially enhanced product selectivity towards active metabolites owing to the partial removal of salts and monosaccharides in the permeate fraction (See Figure 7). 

Although no significant difference in the contents of total tannins and flavonoids was observed, as can be appreciated in Figure 7, NF increased the retentate’s TPC by more than 35% compared to the D-PP extract, although there was also a slight contraction in TAC. Furthermore, the membrane concentration protocol also increased the antioxidant activity of the NF retentate, which reached 554.33 µmolEDTA/gExtr, compared to the initial 529.81 µmolEDTA/gExtr of the D-PP scaled-up extract, and 12.13 vs. 5.72 mmolTE/gExtr. Moreover, the effective recyclability of the aqueous solution recovered in the permeate stream was tested, and it was found that the process negligibly affected the final product quality.

**Figure 8:**
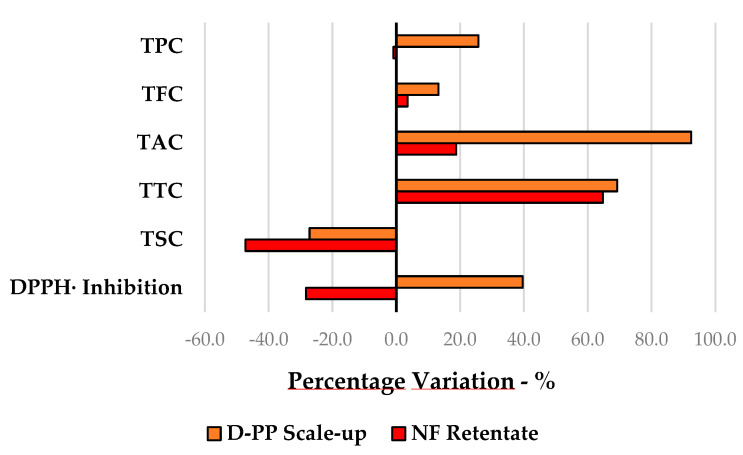
Comparison with hydroalcoholic benchmark extraction for D-PP scaled-up extraction and the related NF retentate. Percentage variations are calculated using the conventional benchmark as the reference (y-axis).

For the sake of comparison, the efficiency of the MW-assisted pilot-scale protocol for D-PP extraction was evaluated against a hydroalcoholic process that was adopted as a benchmark (Section 2.3). The percentage variations for MAE compared to the conventional protocol are depicted in Figure 8, wherein negative percentages represent values superior to those of the benchmark. Simple MAE of D-PP did not outperform the reference, except for TSC and Cu chelating activity values (1.2- and 7-fold, respectively). Nevertheless, the additional downstream NF step dramatically enhanced the value of the final product, overtaking the hydroalcoholic extract’s TPC and DPPH· inhibition values, in the latter by more than 28%. The remaining parameters, as seen in Figure 8, approached those of the reference, after increases in TFC and TTC of 9.7% and 4.5%, respectively.

### 3.3. Biological Activities of PP Extracts

PP extracts have been used in traditional medicine for centuries because of their various health benefits. A wide range of bioactive compounds, including polyphenols, flavonoids, tannins, anthocyanins, and ellagitannins, contribute to their biological activities, which include antioxidant, anti-inflammatory, antimicrobial, and anti-cancer properties [54]. In this work, we assessed the main PP extract’s antimicrobial properties against various bacteria and its anti-cancer activity, in terms of its potential to inhibit the growth of cancer cells. In the above-mentioned tests, we explored the products that were recovered from the optimised protocol and then characterised, including the scaled-up F-PP, D-PP, and D-PP/NF extracts together with the hydroalcoholic benchmark.

#### 3.3.1. Antibacterial Activity of PP Extracts

**Figure 9:**
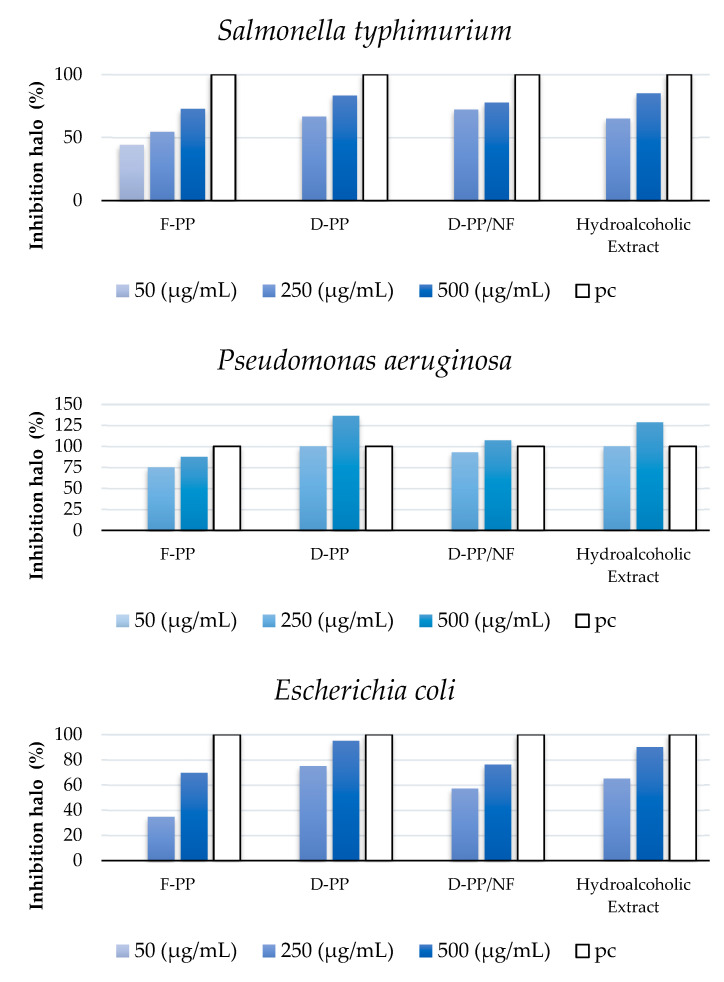

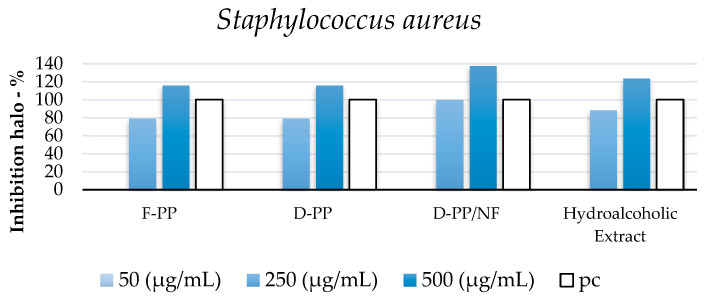
Antimicrobial activity of PP extracts against pathogens (mm). pc = positive control. Reported data are normalised against pc.

**Figure A1:**
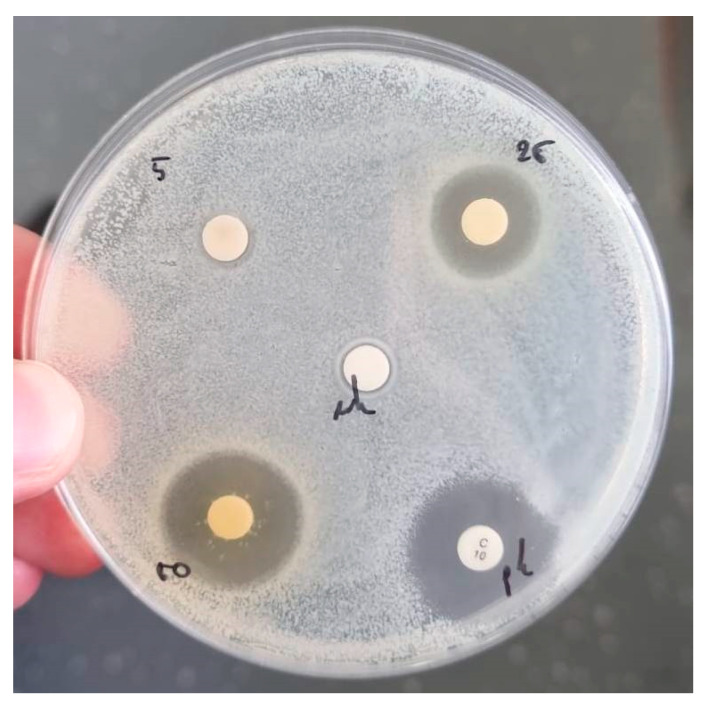
Representative result of a disc diffusion assay on S. aureus treated with PP extract (50, 250, and 500 µg/mL) compared to negative and positive controls.

Antibacterial activity against common Gram-positive (*Staphylococcus aureus*, *Bacillus subtilis*, and *Listeria monocytogenes*) and Gram-negative (*Escherichia coli*, *Pseudomonas aeruginosa*, and *Salmonella typhimurium*) pathogenic bacterial strains was evaluated. Figure 9 shows the activity determined using a disc diffusion assay (example in Figure A1), normalised against the positive control (as 100%). The size of the inhibition halo was proportional to the susceptibility of the organism to the tested compound, meaning that the larger the inhibition zone, the more susceptible the bacteria to a particular extract. Complete data are reported in Appendix A (Table A2).

According to the results presented in Figure 9, the majority of pathogenic strains appeared to be sensitive to the PP extracts to some extent. The exceptions were *Listeria monocytogenes* and *Bacillus subtilis*, since no inhibition halos were detected with any of the four samples tested at any applied concentration (thus they are not reported in Figure 9). Of the tested pathogenic bacteria, only *S. typhimurium* was affected by F-PP at the lowest tested concentration (50 µg/mL), with an inhibition halo of 9.7 ± 0.6 mm. As can be seen in Figure 9, *S. aureus* was the most sensitive to all of the tested samples, as the inhibition halos of the PP extracts at a concentration of 500 µg/mL were 21 ± 0.6 and 22 ± 1.2 mm in size. Moreover, it worth mentioning that the PP extracts showed greater inhibition activity than the positive control. The same effect could be observed at a lower sample concentration (250 µg/mL), for which *S. aureus* was the most inhibited bacteria (15 ± 0.6 to 16 ± 0.7 mm), although there was no significant difference in inhibition compared to the other three Gram-negative bacteria.

The obtained results were comparable to the work by Emam-Djomeh et al. [55], who investigated the antimicrobial activity of PP extracts towards *E. coli* O157:H7 and *S. aureus*. According to their results, *S. aureus* was more sensitive than *E. coli*, as the MIC (minimal inhibitory concentration) and MBC (minimal bactericidal concentration) of the samples for *S. aureus* were 125 and 250 ppm, respectively, while the MIC and MBC for *E. coli* were 250 and 500 ppm, respectively. Differences in cell surface hydrophobicity [56], the higher complexity of the double membrane enveloping Gram-negative bacteria, and the solubility of extracts in the lipid phase of the membrane are some of the factors that have been suggested as being responsible for the differences in the antibacterial activity of PP extracts. In comparison, Gram-positive cells have a single layer of glycoprotein/teichoic acid, and even though it is thicker than in Gram-negative cells, it clearly does not provide enough protection to cells.

Research by Fabrizio Ferrazzano et al. [57] demonstrated that 10 and 15 µg/mL hydroalcoholic PP extracts effectively inhibited the growth and survival of *S. mutans* ATCC 25175 strain and an *R. dentocariosa* clinical isolate. In comparison, Alexandre et al. [58] investigated the antimicrobial effects of by-products obtained via high-pressure enzymatic extraction of pomegranate against five Gram-positive (*B. cereus*, *S. aureus*, *methicillin-resistant S. aureus*, *L. innocua*, and *L. monocytogenes*) and three Gram-negative (*S. enteritidis*, *P. aeruginosa,* and *E. coli*) food pathogenic bacteria and five bacteria used as markers of beneficial gut microbiota, i.e., lactic acid bacteria (LAB) (*L. plantarum*, *L. rhamnosus*, *L. acidophilus*, and two strains of *B. animalis*). All of the tested PP extracts demonstrated selective antimicrobial activity against the pathogenic bacteria without affecting the beneficial ones. Unlike our results shown in Figure 9, Alexandre et al. [58] showed that *P. aeruginosa* was more sensitive to the peel extract than all of the other tested microorganisms. In the case of LAB, only small inhibition halos were observed for *L. rhamnosus* and *L. acidophilus*, which was related with the fact that LAB can metabolise phenolic compounds into volatile phenols, whereas the sugar moieties of anthocyanins may be used as an energy source [59,60]. Furthermore, the research by Alexandre et al. [58] and Dahham et al. [61] showed that *B. cereus*, *E. coli*, *S. aureus*, and *P. aeruginosa* were affected by PP extract. Ismail et al. also reported that this product displayed inhibitory effects against *B. subtilis*, *S. aureus*, *P. aeruginosa*, *E. coli*, and *S. typhimurium* [62]. However, our results were not in complete agreement with those of Ismail et al. (2016) and Alexandre et al. (2018), because *B. subtilis* and *L. monocytogenes* were not affected by the tested extract in our work. The observed differences in activity in different studies may be partially explained by variations in the phenolic contents of the extracts, the antimicrobial procedures adopted in the tests, and strain sensitivity, as well as by differences in the strains used.

#### 3.3.2. Antiproliferative Activity of PP Extracts

**Figure 10:**
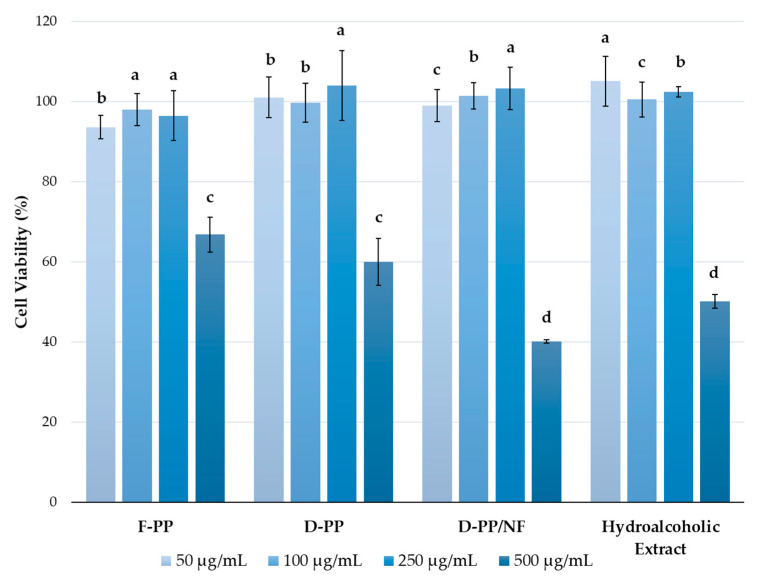
Viability of HeLa cells treated with four PP extracts for 72 h in the concentration range from 50 to 500 μg/mL, assessed using the CellTiter AQueous One Solution Cell Proliferation Assay. Cell viability (%) is expressed as percentage of treated cells versus control cells. Presented value followed by different lower-case letters (a–d) are significantly different from each other in each group (*p* < 0.05), as measured by Tukey’s HSD test.

The antiproliferative activity of four PP extracts (scaled-up F-PP, D-PP, and D-PP/NF extracts together with the hydroalcoholic benchmark) was evaluated in experiments that investigated their ability to inhibit the growth of HeLa carcinoma cells. The cytotoxicity of the prepared samples was measured using the CellTiter AQueous One Solution Cell Proliferation Assay. The results were expressed as the cell viability (%) of treated cells against controls (non-treated cells), as shown in Figure 10.

All of the tested extracts displayed antiproliferative activity when cells were treated with the highest concentration of extract (500 μg/mL), with the most pronounced effects exerted by D-PP/NF with a cell viability of 40.21%. On the other hand, the lowest impact on cell growth was observed with F-PP, with 66.84% cell viability after 72 h of treatment at the highest tested concentration. Similar activity has already been reported in the literature, mostly for ethanolic PP extracts, and in some cases, it was related to cell death as well as cell cycle arrest. For example, Keta et al. investigated the effects of an aqueous-ethanolic PP extract on various human cancer cell lines (HTB140, HTB177, MCF7, and HCT116) at a broad range of concentrations (15.63, 31.25, 62.5, 125, 250, 500, and 1000 μg/mL) [63]. They observed that the samples obtained using 50% ethanol in a US bath expressed selective cytotoxicity to cancer cells. Three cancer cell lines (HTB177 < HCT116 < MCF7) were considered to be sensitive to the obtained product, with MCF7 cells being the most sensitive. Work by Keta et al. [63] reported that a cytotoxic effect was observed at a lower concentration than that used in our work, with this difference possibly being related to the use of different types of extraction procedures and, therefore, different profiles of biologically active substances in the products. Similar results were observed with a methanolic extract of PP by Modaeinama et al. [64]. Cytotoxicity (0, 5, 20, 100, 250, 500, and 1000 μg/mL) was evaluated in MTT assays on lung non-small cell cancer (A549), breast adenocarcinoma (MCF-7), ovarian cancer (SKOV3), and prostate adenocarcinoma cells (PC-3). The samples reduced cell viability to values below 40%, even at the lowest doses, with EC_50_ values below 5 μg/mL, which was significantly lower than those observed here in HeLa cells. MCF-7 breast adenocarcinoma cells were the most responsive to the antiproliferative effects of PP extracts, as reported by Keta et al. [63]. The potential anti-cancer activity of PP extracts on the growth and cell death mechanisms of chronic myeloid leukaemia (CML) cells (K562) was also investigated by Asmaa et al., who observed that ethanolic samples inhibited the growth of K562 cells, mainly via cell cycle arrest and apoptosis induction, but at a lower rate [65]. One recent in vitro study by Nasr et al., performed against the liver cancer cell line (HepG2), confirmed the dose-dependent cytotoxicity of the tested extracts, which led to cell cycle arrest and cell death [66]. The same group also compared the extracts of pomegranate seed and peels, and demonstrated that cell inhibition was much more effective by the former. A recently published study investigated the potential antiproliferative effect of an extract of whole pomegranate fruit on the human breast cancer line (AU565-PAR) and peripheral blood mononuclear cells (PBMC) from healthy donors. The effect on the growth of healthy PBMC cells was observed at a higher concentration than that used in our study (EC30 ≥ 50 mg/mL) [32]. Therefore, the extract from D-PP /NF, as well as others that showed cytotoxicity towards HeLa cells, could be expected to have no negative effects on healthy cells, which should certainly be further investigated before a possible commercial application of such extract.

**Figure 11:**
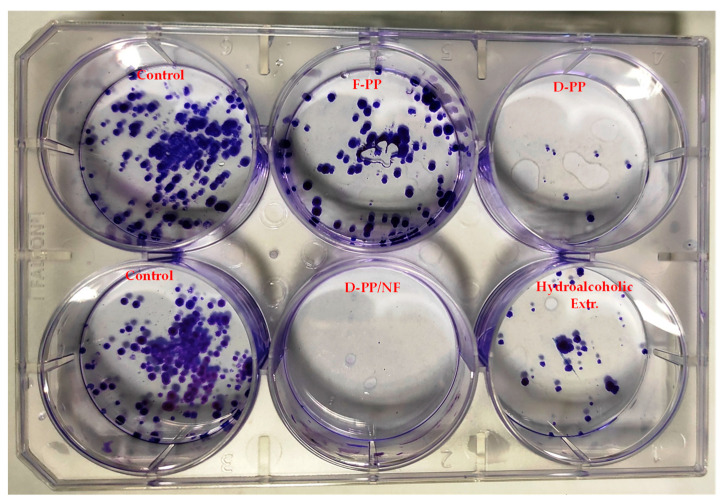
Results of clonogenic analysis after treatment with PP extracts at a concentration of 500 µg/mL.

In order to deepen our knowledge of the potential anti-cancer activity of the explored samples, the “colony formation test” or clonogenic assay was exploited in this work as another in vitro method. It is based on the ability of a single cell to grow into a colony. Clone formation is, in some way, a property of unlimited growth, which is a special feature of tumor cells, meaning that the clonogenic assay could serve as a good indicator of the antitumor potential of the tested compounds. The effects of the PP extracts were analysed on HeLa cells treated with 500 μg/mL of the samples, as indicated in Figure 10. After 19 days of in vitro cultivation, colonies became visible and were then coloured with 0.5% crystal violet, counted, and photographed, as shown in Figure 11. 

Based on the number of colonies counted, the plating efficiency (PE) and surviving fraction (SF) were calculated for the tested compounds (Table 4), according to the equations in the protocol reported by Franken et al. [45]. A higher SF value meant that a higher colony-forming ability was maintained after treatment with the extract, with this ability potentially being related to that compound having less pronounced cytotoxic efficacy.

Table 4 shows the SF values of the treated HeLa cells. A higher value here (~1) indicated higher colony-formation ability after treatment. It was evident that there were differences in the SF values of the four tested extracts. The colony-formation ability depended on the type of sample extract used, with D-PP/NF seeming to be the most potent in terms of anti-cancer activity, as evidenced by its SF value of zero. The SF values for F-PP and the hydroalcoholic extract were higher than those for the other two evaluated compounds, which was consistent with their weaker inhibitory effect in the antiproliferative assay. Clonogenic analysis was also performed by Keta et al., but with significantly lower PP extract concentrations (16 μg/mL), resulting in an SF of 0.74 in HTB177 cells, which were found to be more resistant than HTB140 cells that displayed an SF of 0.47 [63]. In their work, no colony formation was observed after treatment with 32 μg/mL of extract, indicating that clonogenic growth was completely inhibited. 

It could be concluded from the results of the antiproliferation assay and clonogenic analysis with the HeLa cell line that the tested PP extract exhibited potential to act as a natural anti-cancer product with biological activity. Other studies have also demonstrated that similar samples can inhibit the proliferation of cells in various types of cancer, including breast, prostate, colon, lung, and skin cancers. In particular, PP extracts’ bioactive compounds have been found to induce cell cycle arrest, decrease cell viability, and promote apoptosis (programmed cell death) [54,63,65,66] through several mechanisms of action, such as antioxidant effects, anti-angiogenic effects, and the modulation of signalling pathways, as well as showing chemopreventive potential.

### 3.4. Green Metrics and Power Consumption of Extraction: A Preliminary Evaluation

Green metrics play a crucial role in evaluating environmental impact, serving as valuable tools to assess the efficiency and sustainability of chemical processes. Three of the main green metrics, RME, E-Factor, and PMI (PME), were exploited to evaluate the proposed MAE approach for D-PP valorisation followed by the NF step, adopting the hydroalcoholic benchmark protocol as a reference. The values obtained using Equations (3)–(6) (Section 2.7) are reported in Table 5. 

First, RME can be used to describe how the matrix is exploited by the process, thus generating lower amounts of waste (see Equation (3)). In this case, the conventional protocol showed the best result, with an approx. 7% gap with the enabling technologies. However, RME does not consider solvents and their involvement in waste generation. For this reason, the E-Factor was considered since it includes solvents in the calculation (see Equation (4)). It worth remembering that lower values indicate a more sustainable process with minimal waste generation. According to the E-Factor values, the process intensification of MAE and MAE/NF was dramatically more environmentally friendly than the conventional protocol, although the E-Factor can provide a bias in the evaluation of aqueous extractions as it excludes water. Hence, PMI and the more intuitive PME were considered to be the most objective green metrics (see Equations (5) and (6)) [22]. In this evaluation, the conventional and MW-assisted processes were too close to call (only 0.42% difference), whilst the MAE-NF strategy was found to be the most sustainable protocol, as it more than doubled the PME value of the hydroalcoholic process. This result could be easily explained by the capacity to recycle approx. 75% of the overall amount of water exploited.

The utilisation of green metrics plays a pivotal role in providing a guide for the design and optimisation of sustainable processes. More environmentally viable practices can be adopted, achieving the objectives of waste minimisation, resource conservation, and reduction of the overall environmental impact associated with chemical processes.

Power consumption, already assessed in Section 3.2.2 (D-PP/F-PP comparison), was a rational addition to the green metrics evaluated so far, and so was tentatively extended to MAE scale-up/NF. The whole process was considered, including downstream elements, when evaluating the overall consumption and relative power distribution of each step (see Figure 9).

**Figure 12:**
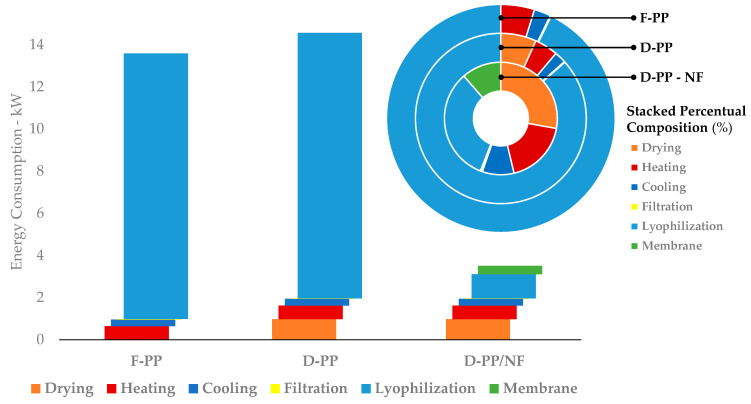
Power consumption and relative distribution. All data refer to scaled-up extractions.

As expected, the lyophilisation procedure had the largest contribution to the global power intake, for both dry and fresh peels. On the other hand, this factor could be dramatically reduced thanks to concentration of the extract, performed using NF, in the case of D-PP. Furthermore, this strategy reduced the power consumption by more than 4-fold (3.51 vs. 14.57 kW). As depicted in Figure 12 (donut chart detail), membrane filtration was an extremely energy-efficient solution, accounting for a minimal part of the requirements of the whole process. Moreover, its viability for industrial applications is already established, supported by the availability of several NF systems accessible on the market, resulting in high flexibility and reuse, in consideration of limited resource consumption (washing/regeneration).

Obviously, the removal of intake water can also be considerably decreased using other approaches, such as spray drying systems. However, despite possessing a lower energy/evaporation ratio, this technology usually requires excipients to maintain good product recovery, which reduces metabolite selectivity and, thus, extract activity. Lastly, it was possible to state that, in general, the concentration of extracts by NF had beneficial effects compared to every type of dewatering technique. In fact, it was possible to refine the *Polyphenol Power Efficiency* calculation, obtaining 277.32, 82.92, and 20.92 W/mgGAE, respectively, for F-PP, D-PP, and D-PP/NF. Thus, it was possible to state that with the same amount of power, the polyphenol productivity could be approximately quadrupled in the case of the MW-assisted PP-valorisation process followed by an NF step.

## 4. Conclusions

Pomegranate (*Punica granatum* L.) production is estimated to be about 8.1 million t/y, with the related peel generation being approx. 3.6 million tons, making it a huge environmental problem and economic loss. In this work, an MAE protocol was optimised by evaluating the recovery of several components, such as polyphenols, anthocyanins, flavonoids, tannins, and sugars, and their antioxidant and Cu chelating activities.

During the screening, the matrix nature was explored in addition to time and temperature variations, as we investigated how product quality and yield were affected by fresh and dried peels, and the latter was found to be the most promising feedstock. This finding would help to reduce the overall mass of matrix to be fed into the extraction system, confirming adequate productivity and even enhancing the quality of the product. Furthermore, avoiding fresh material could slim down the production chain, cutting storage and transportation costs. The study also involved the evaluation of potential water recycling, performed by means of a nanofiltration approach, which led to overall water reuse of approx. 75%. An evaluation of power consumption (20.92 W/mgGAE) and common green metrics gave encouraging results in terms of the sustainability of the non-conventional valorisation strategy vs. the conventional approach (PME: 5.55% and 2.26%, respectively).

The screening also involved a preliminary transposition from lab-scale to a scaled-up protocol, from 1 to 20 g, as a feasibility test, paving the way for further piloting considerations in view of the enormous amount of waste produced yearly. The achieved results showed promising outcomes, paving the way for further piloting considerations, considering the increasing TRL of MW.

The product provided by the tuned extraction protocol boasted the following contents per gram of PP extract: TPC: 356.35 mgGAE; TAC: 303.97 µgCy3G; TFC: 37.28 mgQE; TTC: 56.48 mgGAE; and TSC: 615.41 mgGLU. In addition, the Cu chelating activity was 529.81 µmolEDTA and the antioxidant activity was 5.72 mmolTE (according to the adopted reference). The biological activity of the recovered PP extracts was also studied. The samples possessed antimicrobial activity in laboratory studies, but further research is needed to determine their effectiveness for potential applications in the food, cosmetics, and even pharmaceutical industries. While the findings on the extracts’ antiproliferative activity, i.e., the anti-cancer activity of the PP extracts, were also promising, it is important to note that research on the anti-cancer activity of pomegranate extracts is still evolving, and more studies, including clinical trials, are needed to determine their efficacy, optimal dosage, and potential interactions with other cancer therapies. Nevertheless, pomegranate peels are a valuable by-product and their extracts, especially those achieved using green extraction methods, deserve further investigation du to the numerous benefits of the approach. The present manuscript, with all of the gathered data and the extraction/downstream/characterisation approach, contribute to filling in the lack of research on MAE of PP, according to previous work and literature reviews.

## Figures and Tables

**Table 1:** Thermogravimetric analysis of F-PP and D-PP.

Fraction	Percentage Composition (%)	
	Fresh Matrix	Dried Matrix
Water	75.52	3.50
Organic	23.86	94.06
Inorganic	0.62	2.44

**Table 2:** Antioxidant activity expressed as DPPH· radical inhibition and Cu chelating activity, with relative EC50, Trolox eq. and EDTA eq. values. Samples produced at 100 °C, 10 min.

	DPPH· Inhibition		Cu Chelating Activity	
Matrix	EC50	Trolox eq.	EC50	EDTA eq.
	(µg/mL)	(mmolTE/gext)	(µg/mL)	(µmolEDTA/gext)
F-PP	2.30 ± 0.45	13.18 ± 2.58	88.60 ± 9.8	513.66 ± 58.8
D-PP	5.30 ± 1.45	5.72 ± 1.57	85.90 ± 13.6	529.81 ± 83.9

**Table 3:** Scaled-up MAE (100 °C, 10 min) of F-PP and D-PP: polyphenol power efficiency calculated as a function of yield and power consumption.

	D-PP	F-PP
Dry yield (%)	67.20	20.18
TPC Yield (mgGAE/gMatrix)	175.64	48.99
Power Consumption (kW) *	1.94 **	0.97
**Polyphenol Power Efficiency (W/mgGAE)**	**11.07**	**19.72**

* Power required by the scaled-up protocol to heat and cool the MW reactor; ** Power required for the drying procedure is added for D-PP.

**Table 4:** Surviving fraction (SF) of HeLa cells treated with PP extracts (500 µg/mL).

	F-PP	D-PP	D-PP/NF	Hydroalcoholic Extr.
SF	0.646	0.074	0	0.274

**Table 5:** Green metrics for MAE of D-PP: comparison of hydroalcoholic benchmark, D-PP Scale-up extract, and its relative NF extract. Green bars: metrics considered directly proportional to sustainability; red bars: metrics considered inversely proportional to sustainability.

	RME (%)	E-Factor	PMI	PME (%)
Hydroalcoholic Benchmark	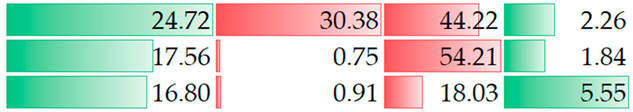			
D-PP Scale-up				
D-PP Scale-up NF				

## Data Availability

No new data were created or analyzed in this study. Data sharing is not applicable to this article.

## Appendix A

**Table A1:** PP extraction screening: dry extraction yields for fresh and dry matrices.

Matrix	t (min)	T (°C)	Dry Yield (%)	S.D. (%)	
Fresh	10	100	20.18	±	0.37
	20		22.19	±	0.56
	30		21.38	±	0.60
	10	125	22.38	±	0.71
	20		22.39	±	0.42
	30		21.49	±	0.74
	10	150	22.71	±	0.58
	20		23.52	±	0.31
	30		26.40	±	0.85
Dried	10	100	67.20	±	0.79
	20		67.40	±	1.14
	30		65.00	±	1.19
	10	125	68.99	±	0.82
	20		75.57	±	0.78
	30		74.80	±	1.00
	10	150	73.43	±	0.98
	20		73.79	±	0.83
	30		74.85	±	1.48

**Table A2:** Antimicrobial activity of PP extracts against pathogens (mm). pc = positive control; nc = negative control.

	Conc.	*S.* *typhimurium*	S.D.	*P.* *aeruginosa*	S.D.	*E. coli*	S.D.	*S. aureus*	S.D.	*L.* *monocytogenes*	S.D.	*B.* *subtilis*	S.D.
	(µg/mL)												
F-PP	50	9.7	0.6	0	0	0	0	0	0	0	0	0	0
	250	12	0.6	12	0.6	8	2.1	15	0.6	0	0	0	0
	500	16	1.5	14	1	16	1.5	22	1.2	0	0	0	0
	pc	22	1.2	16	1.2	23	0.6	19	1	17	0	16	1.2
	nc	0	0	0	0	0	0	0	0	0	0	0	0
D-PP	50	0	0	0	0	0	0	0	0	0	0	0	0
	250	12	0	11	1.2	15	3.1	15	0.6	0	0	0	0
	500	15	1.2	15	2.1	19	1.2	22	1.2	0	0	0	0
	pc	18	3.5	11	1.2	20	1	19	1	16	2.5	17	0
	nc	0	0	0	0	0	0	0	0	0	0	0	0
D-PP/NF	50	0	0	0	0	0	0	0	0	0	0	0	0
	250	13	0	13	1.2	12	1	16	0.7	0	0	0	0
	500	14	0.6	15	1.2	16	2.1	22	1.2	0	0	0	0
	pc	18	0.6	14	1	21	1	16	0.6	15	0.6	16	1
	nc	0	0	0	0	0	0	0	0	0	0	0	0
Hydroalcoholic Extract	50	0	0	0	0	0	0	0	0	0	0	0	0
	250	13	0.6	14	0	13	1.2	15	0.6	0	0	0	0
	500	17	1	18	0.6	18	1.5	21	0.6	0	0	0	0
	pc	20	1	14	2.1	20	0	17	1	14	1.5	12	0.6
	nc	0	0	0	0	0	0	0	0	0	0	0	0
